# Persistent organic pollutants in pregnant women potentially affect child development and thyroid hormone status

**DOI:** 10.1038/s41390-021-01488-5

**Published:** 2021-04-06

**Authors:** Anna A. Krönke, Anne Jurkutat, Maike Schlingmann, Tanja Poulain, Matthias Nüchter, Anja Hilbert, Hannu Kiviranta, Antje Körner, Mandy Vogel, Olle Söder, Carl G. Bornehag, Wieland Kiess

**Affiliations:** 1 Department of Women and Child Health, Hospital for Children and Adolescents and Center for Pediatric Research (CPL), Leipzig University, Leipzig, Germany; 2 LIFE Leipzig Research Center for Civilization Diseases, University of Leipzig, Leipzig, Germany; 3 Integrated Research and Treatment Center Adiposity Diseases, University of Leipzig, Leipzig, Germany; 4 Department of Psychosomatic Medicine and Psychotherapy, University of Leipzig, Leipzig, Germany; 5 Department of Health Security, National Institute for Health and Welfare (THL), Kuopio, Finland; 6 Pediatric Endocrinology Unit, Department of Women’s and Children’s Health, Karolinska Institute, Stockholm, Sweden; 7 Public Health Sciences, Karlstad University, Karlstad, Sweden; 8 Icahn School of Medicine at Mount Sinai, New York, NY USA

## Abstract

**Background:**

Potentially harmful effects of persistent organic pollutants (POPs) such as polychlorinated biphenyls (PCBs) and dichlorodiphenyltrichloroethane (DDT) on prenatal development and the endocrine system have been controversially discussed.

**Methods:**

Working with a German cohort of 324 pregnant women, we assessed POP levels and used robust linear regression models to determine potential associations between maternal POP concentrations and pre- and postnatal development in the children, as well as the thyroid hormone status of the mother and child.

**Results:**

Maternal *p*,*p*′-dichlorodiphenyldichloroethylene (*p*,*p*′-DDE) and most measured PCBs positively correlated with postnatal weight gain. We detected no correlation between newborn birth weight and head circumference, respectively, and maternal PCB and *p*,*p*′-DDE serum levels, while body length at birth was negatively associated with the maternal serum concentration of PCB 183. Maternal *p*,*p*′-DDE and nearly all PCB serum levels showed a negative correlation with maternal free triiodothyronine (FT3). *p*,*p*′-DDE and PCB 74 and 118 were negatively associated with maternal thyroid-stimulating hormone levels. In addition, we identified significant associations between maternal POP levels and thyroid hormone parameters of the child.

**Conclusions:**

These results indicate that POP exposure likely affects different aspects of pre- and postnatal development and impacts the thyroid hormone status of both mother and child.

**Impact:**

- Pregnant women in a German cohort display a substantial accumulation of POPs.
- Body mass index and age influence maternal serum POP levels.
- Maternal POP levels show correlations with the child’s length at birth and weight gain, and FT3 levels in the mother and child.
- Our data provide additional evidence for the potentially harmful influence of POPs.
- Our data indicate that POPs influence pre- and postnatal development.

## Introduction

Polychlorinated biphenyls (PCBs) exhibit varying degrees of halogenation, ranging from one to ten chlorine atoms. As the number of chlorine atoms increases, so does the environmental persistence of the PCB in question.^1^ Due to their chemical properties, PCBs were widely used as coolant and dielectric fluids, in capacitors and as a joint sealing material.^2^ The parties of the Stockholm convention largely banned the production and use of PCBs, along with that of other persistent organic pollutants (POPs) such as the insecticide dichlorodiphenyltrichloroethane (DDT), but an equipment that contains or is contaminated by PCBs may be used until 2025.^3^

Although the implementation of such prohibitive measures has led to a decrease in their environmental concentrations, these pollutants can still be detected in organisms worldwide. The reason for the persistence of these substances is their resistance to environmental degradation and high lipophilicity, which leads to accumulation both in the food chain and in human tissues.^1^ Importantly, there is increasing evidence that they still act as an endocrine-modulating substance, even at low levels that were initially considered safe.^4^ The results of several animal experiments and a number of epidemiological studies suggest that the prenatal phase and childhood are especially vulnerable to the influence of endocrine modulators of this sort.^5^ It is for this reason that—in the publication “State of the science of endocrine disrupting chemicals—2012”—the World Health Organization recommended focusing further investigations on the impact of these compounds on human prenatal development.^5^ There is evidence that prenatal exposure to certain PCBs and to *p*,*p*′-DDT and its main degradation product, *p*,*p*′-dichlorodiphenyldichloroethylene (*p*,*p*′-DDE), can have adverse effects on growth and metabolism, and on neurocognitive development, sexual development and reproduction.^6^

Studies suggest that PCBs and *p*,*p*′-DDT disturb the metabolism through hormone receptor interaction^7^ and subsequent changes in histone methylation.^8, 9^ Resulting changes in the epigenome and transcription rate of certain genes are thought to cause alterations in lipid metabolism and adipogenesis.^10^ Several studies also suggest that *p*,*p*′-DDT and PCBs may impair growth and metabolism through a disturbance of the thyroid gland.^11, 12^

The present study aims to identify demographic determinants of serum concentrations of different PCBs and the organochlorine pesticide *p*,*p*′-DDT in pregnant women in Germany, a country that banned the use and production of these POPs in 1991. Furthermore, this study aims to analyse the association between POP serum concentration in mothers at the 24th or 36th week of pregnancy and anthropometric data for their children at birth. With a view, specifically, to identifying the influence—if present—of prenatal exposure to PCBs and DDE on prenatal growth and metabolism, we assessed foetal growth and development using weight, length, and head circumference at birth, and the duration of pregnancy. We additionally analysed weight gain in the first 2 years of life, as fast weight gain in early childhood and low birth weight have been associated with an increased risk of cardiovascular and metabolic disease in adulthood, in line with the Barker hypothesis.^13, 14^ In addition, we analysed the influence of maternal PCB and *p*,*p*′-DDE serum levels on thyroid hormone parameters such as thyroid-stimulating hormone (TSH), free triiodothyronine (FT3), and free thyroxine (FT4) both of the mother during pregnancy and of the child at 6 months and at 1 year.

## Subjects and methods

### Subjects

The study population consisted of 333 women and their children, who were selected from within the LIFE Child cohort.^15^ LIFE Child is a large, population-based, longitudinal childhood cohort study based in the city of Leipzig in the former East Germany that seeks to monitor healthy child development from birth to adulthood and to offer a more in-depth understanding of the development of lifestyle diseases. The LIFE Child cohort includes mothers born in both the former East Germany and the former West Germany. In the case of pregnant women, assessments were carried out in the 24th and 36th week of pregnancy. Children were assessed at 3, 6, and 12 months of age and once per year thereafter. The mother–child study programme includes age-adapted medical, psychological and sociodemographic assessments and the collection of biological samples.

**Fig. 1:**
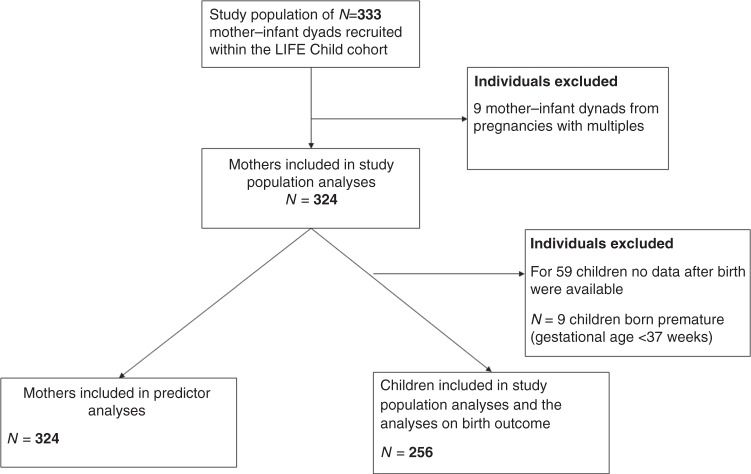
Visualized study population. In certain models, incomplete data in the mentioned booklets or missing data relating to individual covariates led to a further reduction of sample size.

From our cohort of 333 mothers and their children, nine children and their mothers were excluded due to multiple pregnancies. For 59 out of the remaining 324 women, no data about the children after birth were available. Of the children, 3.5% (*n* = 9) were born premature (defined as gestational age <37 weeks). These children were excluded from all analyses, except those who were involved in the length of pregnancy, resulting in a sample size of 324 mothers and 256 children born following full-term pregnancies. (Fig. 1). In the case of certain models, the sample was reduced further where the necessary data were missing in the “Your child’s medical records” or “Maternity Records” booklets (see below), or where data for specific covariates were otherwise unavailable.

The study was designed in accordance with the Declaration of Helsinki. The Ethics committee of the University of Leipzig was involved in the development of the study model and granted its approval for the final design (Reg. No. 264/10-ek). Fully informed, written consent has been obtained from all participants and their parents.^15^

### Outcome parameters

Weight at birth was measured by a midwife directly after birth using a clinical scale. The weight at 1 and 2 years was measured by professional study nurses at the LIFE CHILD study centre using a clinical scale. All anthropometric data, including weight, length and head circumference at birth, were sourced from the booklets “Your child’s medical records”, which is used throughout Germany to record a child’s development, and “Maternity records”, which is used to document the course of the pregnancy. To calculate the pre-pregnancy body mass index (BMI), the pre-pregnancy weight and height were taken from the “Maternity records” booklet.

To determine the social status of the participants, an adjusted socioeconomic index as proposed by Winkler and Stolzenberg^16^ was applied. This index is calculated using the parents’ education level and occupational status and the net equivalent household income, and is expressed in a value between 3 and 21 inclusive, with a score between 3 and 8 indicating low socioeconomic status (SES), a score from 9 to 14 indicating middle SES and values from 15 to 21 was defined as high SES.

We assessed the serum levels of TSH, FT3 and FT4 at 6 months and at 1 year, as well as maternal TSH, FT3 and FT4 serum levels at 24 weeks gestation.

### Chemical analysis of the compounds, thyroid hormone parameters and serum lipids

Serum samples were collected between 2011 and 2016 from a pregnant woman in the 24th or 36th week of pregnancy and sent to the National Institute of Health and Welfare laboratory in Kuopio, Finland in February 2016. Results were received in April 2017. Mothers were required to fast for at least 4 h before blood was drawn. The concentration of 12 POPs, among them ten PCB congeners, *p*,*p*′-DDT and *p*,*p*′-DDE, was measured, reported in pg/ml and subsequently correlated with postnatal outcome parameters in both the mother and child. The specific congeners analysed are summarized in Table 2. The ten PCB congeners exhibited a variety of important characteristics such as coplanar and noncoplanar orientation, different degrees of chlorination and dioxin-like and non-dioxin-like structure, as well as different combinations of chlorine atom positions in the molecule.

Full details of the analysis of the chemical compounds are reported elsewhere. However, to summarize, in the sample preparation of POPs ^13^C-labelled internal standards of each compound were added to the samples. Dichloromethane-hexane was used for extraction. Extracts were cleaned with multilayer silica columns. The eluate was concentrated for gas chromatography-tandem mass spectrometry (GC-MS/MS) analysis (Agilent 7010 GC-MS/MS System, Wilmington, DE).^17^ We did not substitute values of compounds below the detection limit to avoid missing weaker signals.^18^

Cotinine, the primary metabolite of nicotine, was measured in the maternal serum in ng/ml. We used it as a biomarker for smoking during pregnancy, with values <1 ng/ml indicating non-smokers, values between 1 and 10 ng/ml indicating non-smoking but heavily exposed women and values >10 ng/ml indicating active smokers.^19^

Analysis of FT3, FT4 and TSH levels was performed via Elecsys electrochemiluminescence immunoassay using the Cobas modular platform (Roche Diagnostics, Basel, Switzerland) as previously described.^20^

Serum levels of total cholesterol and triglycerides were measured via an enzymatic colour test using the Cobas modular platform (Roche Diagnostics, Basel, Switzerland) as previously described.^21^

### Statistics

First, we examined associations between potential influential factors and the compounds of interest using correlation analysis. Subsequently, in regression models, selected variables that showed a significant association with the dependent variable in bivariate analyses or that have been shown to be influential in former studies were included as covariates in the predictor analysis (Model 1).^22–24^ For the models investigating the association between POPs and the thyroid and growth outcomes, the choice of confounders was based on the construction of a directed acyclic graph. We included maternal nicotine consumption as a covariate in the models for birth weight and postnatal weight gain (Table S1, Model 2d–f), as we saw a significant association between these variables, in addition to the covariates determined by creating a directed acyclic graph.

Analyses were performed for individual congeners as well as for grouped compounds. The grouping of the compounds was conducted on the basis of the grouping described by Meeker and Hauser.^25^ Compounds were included, according to their postulated mode of action, in one of two groups:^25^ Group 1, potentially anti-oestrogenic and dioxin-like (congeners 74, 118, 138, 156 and 170); Group 2, phenobarbital, CYP1A and CYP2B inducers (congeners 99, 53, 180 and 183).

To analyse the relationship between the pre-partum serum concentration of the compounds and maternal characteristics, robust linear regression modelling^26^ was performed with each compound as a dependent variable and maternal body mass index (BMI), age at conception, serum lipids (sum of total cholesterol and triglycerides) and SES, in turn, as an independent variable (Model 1; Table 3).

Associations between maternal serum concentrations of the compounds and child development parameters including weight, length and head circumference at birth, weight development in the first and second year of life and length of pregnancy were also analysed using robust linear regression modelling. Maternal age and lipid concentration (total cholesterol and triglycerides), pre-pregnancy BMI and cotinine were considered as potential covariates. Here, we checked for sex differences by adding a respective sex interaction to the model (Model 2, Supplemental Table S1 (online)). Non-significant interaction terms were removed to comply with the principle of parsimony. Due to the statistical approach and small differences between the means and medians of our variables, we did not log transform any variables. The same modelling approach was applied to the relationship between the child serum levels of TSH, FT3 and FT4 at 6 months and 1 year of age, and the maternal compound concentrations using robust linear regression modelling. Maternal serum lipid concentration was used as a covariate (Model 3, Supplemental Table S2 (online)).

Associations between maternal TSH, FT3 and FT4 and compound levels were determined using robust linear regression modelling with serum lipids, BMI and age as covariates (Model 4, Supplemental Table S3 (online)). Furthermore, we used splines to estimate the effective degrees of freedom for all models concerning the thyroid and growth parameters. We also checked if a term of higher order was necessary by modelling the associations with polynomial and compare them to the linear model using analysis of variance. If the higher-order term resulted in a significant model improvement, the results were changed accordingly. Otherwise, the previous result was kept.

We tested for high influence with DFFITS statistic.^25^ Due to the presence of outliers, we chose robust linear regression modelling.

Statistical significance was assumed for all *p* < 0.05. R 3.5.2 was used for all data analyses.^27^

## Results

### Cohort characteristics

The main characteristics of the cohort are summarized in Table 1. From the 324 included mothers, 23.5% (*n* = 76) were overweight or obese (BMI > 25 kg/m²) before pregnancy. The mean age at conception was 30.3 years. Of the women, 5.6% (*n* = 18) were classified as active smokers based on their cotinine measurements; 57.9% (*n* = 165) of the women were categorized as being of middle SES. Of the women, 3.8% (*n* = 12) were born in the former West Germany and 53.8% (*n* = 169) were born in the former East Germany. Ten women were born after German reunification. One hundred and thirty-one women could not be categorized as having been born in East or West Germany. There were slightly more male children (52.7%) than female children. Of the children, 3.4% (*n* = 9) were born premature, that is, before a gestational age of 37 weeks.

**Table 1:** Study population characteristics as mean ± SD or *N* (%).

	Mean ± SD	*N* = (%)
Maternal characteristics		
Age at conception (years)	30.3 ± 4.31	324
Pre-pregnancy BMI (kg/m²)	23.7 ± 4.69	280
Sociodemographic index score^a^		285
Low social status		17 (5.96%)
Middle social status		165 (57.9%)
High social status		103 (36.1%)
TSH (mU/l)^b^	1.83 ± 1.41	268
FT3 (pmol/l)^b^	4.24 ± 0.51	259
FT4 (pmol/l)^b^	12.66 ± 2.07	261
Child characteristics		
Sex child		256
Male		135 (52.7%)
Female		122 (47.7%)
Bodyweight at birth (g)	3538 ± 478.78	256
Gain of bodyweight in the first year (%)^c^	170 ± 38.74	203
Body length at birth (cm)	50.5 ± 2.41	255
TSH (mU/l)^d^ at 1 year	3.15 ± 1.77	93
FT3 (pmol/l)^d^ at 1 year	7.00 ± 0.82	89

### Concentration and correlation of the compounds in maternal serum

**Fig. 2:**
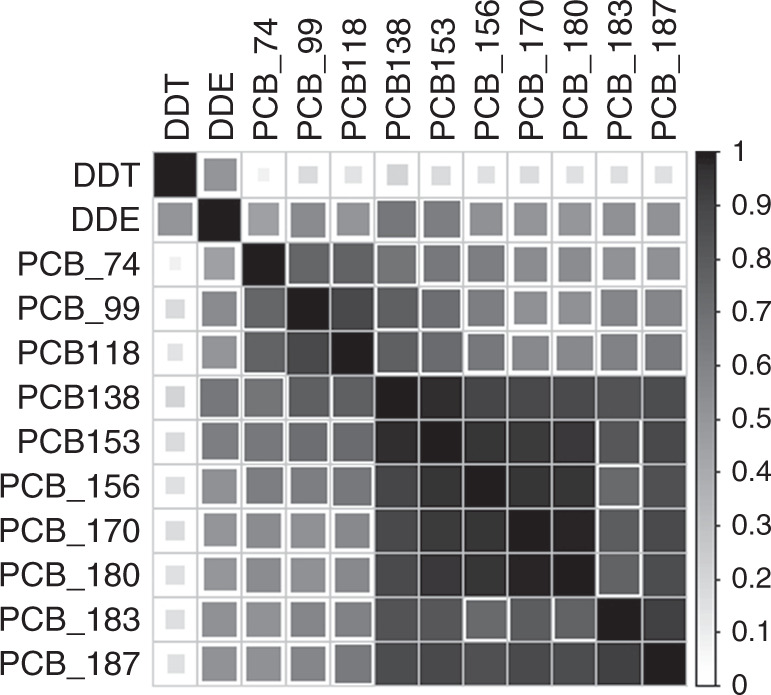
Visualized correlation matrix of the PCB, *p*,*p*′-DDT and *p*,*p*′-DDE serum levels. Correlation matrix with darker shades of grey indicating stronger positive correlations between the compounds. PCB polychlorinated biphenyls, DDT dichlorodiphenyltrichlorethane, DDE dichlorodiphenyldichloroethylene.

Measurement of the maternal PCB and *p*,*p*′-DDT/*p*,*p*′-DDE serum levels revealed varying levels ranging from 7.91 pg/ml for PCB 74 to 128.7 pg/ml for PCB 153. The mean serum level for *p*,*p*′-DDE was 558.73 pg/ml. For most compounds (excluding *p*,*p*′-DDT and PCB 74), the measured serum concentration was above the certified reference concentration (Table 2). The data for all compounds were right skewed. To visualize the correlations between the maternal concentrations of the different individual compounds, we used a correlation matrix (Fig. 2). As expected, the matrix showed correlations between all compounds, with particularly strong correlations between *p*,*p*′-DDT and *p*,*p*′-DDE. Furthermore, we found that the level of correlation between different PCBs was dependent on the degree of chlorination.

**Table 2:** Concentrations of compounds of interest in maternal serum (pg/ml), *N* = 324.

Compounds (pg/ml)	Mean ± SD	Median	%>LOQ	Degree of chlorination
PCB 74	7.91 ± 4.49	6.41	66.05	4
PCB 99	10.01 ± 5.51	8.77	84.26	5
PCB 118	21.22 ± 12.01	18.84	99.70	5
PCB 138	81.66 ± 42.33	75.08	100	6
PCB 153	128.70 ± 69.01	115.69	100	6
PCB 156	13.22 ± 8.06	11.68	91.67	6
PCB 170	44.02 ± 26.10	38.73	100	7
PCB 180	82.85 ± 49.16	71.27	89	7
PCB 183	12.00 ± 6.96	10.52	89.51	7
PCB 187	19.62 ± 12.58	16.31	97.53	7
*p*,*p*′-DDT	23.01 ± 24.56	15.00	29	
*p*,*p*′-DDE	558.73 ± 439.62	417.63	100	

Mean/median serum concentrations of PCB congeners and mean serum concentrations of *p*,*p*′-DDT and *p*,*p*′-DDE are shown. In addition, the respective standard deviation and percent above the lower limit of detection (LOQ) are given. %>LOQ shows the percentage of observations above the certified reference concentration for the compounds in maternal serum (LOQ).

### Demographic determinants of POP levels and associations between POPs and child characteristics

**Fig. 3:**
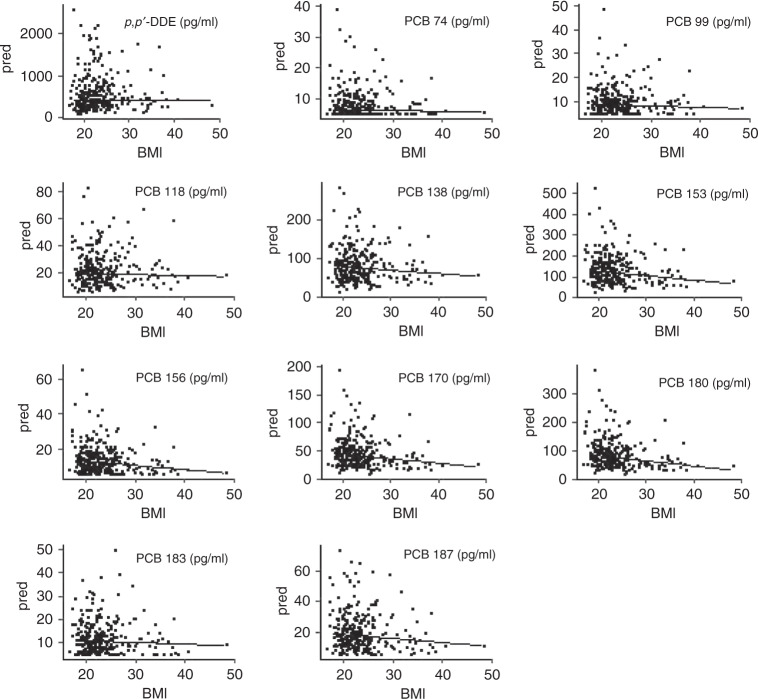
Association between maternal BMI and compound serum concentration. The underlying model for the graph is a robust linear regression model with the individual compound as a dependent variable and maternal age, BMI and socioeconomic score as independent variables. The graphs show the predicted value for the concentration of the compound given a set age and socioeconomic status, while BMI rises in discrete steps between 18 and 48.

Our first aim was to investigate demographic determinants for serum levels of the various POPs measured. Therefore, we created a robust linear regression model with the compounds as a dependent variable and maternal age, BMI, SES and serum lipids as independent covariables. We found significant positive associations between maternal age and serum concentrations for *p*,*p*′-DDE and all PCBs, except PCB 74. Conversely, we observed a negative association between maternal pre-pregnancy BMI and serum concentrations of a subset of PCBs (Table 3). Figure 3 shows the association between BMI and the various POPs in the form of predicted compound serum concentrations for a rising maternal BMI. There were no significant associations between categories of SES and serum levels of *p*,*p*′-DDE or the different PCBs. All measured compounds were positively associated with serum lipid concentrations in the mother.

**Table 3:** Results from robust linear regression models for PCB and *p*,*p*′-DDE maternal serum concentrations (*N* = 246).

Dependent variable	Independent variable	Beta	Std. error	Pr(>|*t*|)
DDE	Age	11.599	4.348	0.008
	SES	6.230	6.412	0.332
	BMI	0.031	3.477	0.993
	Serum lipids	23.995	8.987	0.008
PCB 74	Age	0.071	0.047	0.129
	SES	0.037	0.064	0.564
	BMI	−0.024	0.034	0.481
	Serum lipids	0.302	0.098	0.002
PCB 99	Age	0.158	0.069	0.024
	SES	0.120	0.101	0.237
	BMI	−0.057	0.055	0.295
	Serum lipids	0.607	0.151	<0.0001
PCB 118	Age	0.696	0.173	<0.0001
	SES	0.480	0.263	0.070
	BMI	−0.073	0.144	0.613
	Serum lipids	1.896	0.383	<0.0001
PCB 138	Age	2.649	0.546	<0.0001
	SES	0.702	0.799	0.381
	BMI	−0.691	0.434	0.113
	Serum lipids	7.101	1.141	<0.0001
PCB 153	Age	5.362	0.811	<0.0001
	SES	0.931	1.192	0.435
	BMI	−1.828	0.643	0.005
	Serum lipids	10.632	1.689	<0.0001
PCB 156	Age	0.580	0.089	<0.0001
	SES	0.087	0.131	0.505
	BMI	−0.234	0.071	0.001
	Serum lipids	1.170	0.184	<0.0001
PCB 170	Age	2.307	0.290	<0.0001
	SES	0.122	0.417	0.771
	BMI	−0.818	0.223	0.0003
	Serum lipids	3.811	0.591	<0.0001
PCB 180	Age	4.751	0.508	<0.0001
	SES	0.242	0.727	0.739
	BMI	−1.745	0.391	<0.0001
	Serum lipids	6.444	1.029	<0.0001
PCB 183	Age	0.477	0.099	<0.0001
	SES	0.117	0.146	0.421
	BMI	−0.108	0.078	0.167
	Serum lipids	0.912	0.213	<0.0001
PCB 187	Age	0.966	0.160	<0.0001
	SES	0.049	0.237	0.837
	BMI	−0.298	0.126	0.019
	Serum lipids	1.227	0.349	0.0005

The multivariate regression models revealed consistently negative associations between the compounds and maternal BMI, which reached the level of significance for PCB 153, 156, 170, 180 and 187. There were also consistently positive associations between the compounds and maternal age and maternal serum lipid levels. In this model, we included the compounds of interest as a dependent variable and maternal age, BMI, socioeconomic status and serum lipids as covariates.

*Serum lipids* as the sum of total serum cholesterol and serum triglycerides in mmol/l, *SES* socioeconomic status of participants determined using the adjusted socioeconomic index as set out by Winkler, *Pr(>|t|)* *p* value for the *t* test, *Std. error* standard error, *PCB* polychlorinated biphenyl, *DDE* dichlorodiphenyldichloroethylene.

Next, we analysed associations between maternal PCB and *p*,*p*′-DDE serum levels and child characteristics (Supplemental Table S1 (online)). Maternal levels of *p*,*p*′-DDE and PCBs showed no statistically significant association with the parameters of child weight (g) and head circumference (cm) at birth or with the duration of pregnancy (days). However, we found a significant negative association between the concentration of PCB 183 and length at birth (cm). In addition, concentrations of maternal serum *p*,*p*′-DDE and several PCBs (PCB 99, 118,138, 153, 156, 170, 180 and PCB Groups 1 and 2) positively correlated with weight gain in the first 2 years of life (%). Concentrations of PCB Group 1, which are known to exhibit potentially anti-oestrogenic and dioxin-like “effects”, were also associated with weight gain within the first year of life (Supplemental Table S1 (online)). An analysis of weight gain in the children over the first 2 years of life showed that the weight gained in the children of mothers from the 90th percentile of PCB Group 1 serum concentrations equated to 113% of that gained by children of mothers from the 10th percentile (Table 4).

**Table 4:** Estimates for the weight gain of the child in the first 2 years of life (%) for the 10th, 50th and 90th percentile of maternal serum compound concentration.

Independent variable	Weight gain of child in the first 2 years of life (%) for the		
	10th compound percentile	50th compound percentile	90th compound percentile
DDE	246.264	250.530	264.705
PCB 74	251.391	252.585	258.718
PCB 99	240.943	251.263	271.646
PCB 118	242.247	251.641	270.086
PCB 138	239.655	251.639	273.080
PCB 153	241.322	251.720	271.057
PCB 156	242.808	251.897	270.864
PCB 170	242.596	251.732	270.023
PCB 180	244.247	251.590	269.274
PCB 183	242.654	252.322	270.402
PCB 187	244.326	252.066	268.839
PCB Group 1^a^	240.094	251.498	271.439
PCB Group 2^b^	241.918	251.464	270.269

Robust linear regression models with the child’s weight gain in the first 2 years of life (%) as a dependent variable and the respective compound of interest as an independent variable reveals higher weight gain for higher compound concentrations. Analyses were adjusted for maternal age, maternal BMI, maternal nicotine consumption and maternal serum lipid concentrations.

The underlying model reached the level of significance for DDE, PCB 99, 118, 138, 153, 156, 170, 180 and both PCB groups.

Weight gain in the first 2 years of life (%) = (weight at 2 years (g) − weight at birth (g))/weight at birth (g) × 100.

*PCB* polychlorinated biphenyl, *DDE* dichlorodiphenyldichloroethylene.

^a^Group 1: sum of potentially antiestrogenic and dioxin-like PCB congeners (74 + 118 + 138 + 156 + 170).

^b^Group 2: sum of phenobarbital, CYP1A and CYP2B inducing PCB congeners (99 + 153 + 180 + 183).

We adjusted for maternal age, BMI and serum lipids in the models analysing the maternal thyroid parameters, whereas adjustment for maternal serum lipids was performed in the models analysing the child’s thyroid parameters. Here, we identified significant associations between maternal POP levels and thyroid hormone parameters in both mother and child. Among these, concentrations of maternal PCB 99 and 183 were positively associated with the child’s thyroid hormone parameter FT3 at the age of 6 months for girls only. The mean FT3 level at age 6 months for girls was 6.8% higher in the children of mothers in the 90th percentile for serum concentrations of PCB 99 compared to children of mothers in the 10th percentile (Table 5). Maternal *p*,*p*′-DDE concentrations were negatively associated with FT3 serum concentrations of male children at age 1 year. Maternal *p*,*p*′-DDE, all measured PCB serum concentrations, except PCB 156, and both compound groupings negatively correlated with serum FT3 levels in the mothers. The mean FT3 concentration in mothers in the 90th percentile for *p*,*p*′-DDE serum concentration was 8.1% lower than in mothers in the 10th percentile (Table 6). Table 6 shows the estimates for maternal FT3 for the 10th, 50th and 90th percentile for levels of all congeners. There was an additional negative correlation between maternal serum *p*,*p*′-DDE, PCB 74 and 118 concentration and maternal TSH (Supplemental Table S3 (online)). In line with that, we found a negative correlation between maternal serum PCB 99 and 118 and the child’s TSH levels.

**Table 5:** Estimates for the FT3 serum concentration in the child at age 6 months for the 10th, 50th and 90th percentile of maternal serum compound concentration.

Independent variable	Child FT3 serum concentration at age 6 months (pmol/l) for the		
	10th compound percentile	50th compound percentile	90th compound percentile
DDE female	6.814	6.762	6.783
DDE male	7.216	7.123	6.959
PCB 74 female	6.822	6.845	6.766
PCB 74 male	7.172	7.424	6.962
PCB 99 female	6.489	6.802	7.049
PCB 99 male	7.278	7.10	6.912
PCB 118 female	6.812	6.822	6.747
PCB 118 male	7.188	7.093	7.063
PCB 138 female	6.490	6.820	7.004
PCB 138 male	7.329	7.101	6.871
PCB 153 female	6.512	6.804	7.014
PCB 153 male	7.335	7.111	6.843
PCB 156 female	6.655	6.771	6.915
PCB 156 male	7.321	7.117	6.824
PCB 170 female	6.659	6.752	6.897
PCB 170 male	7.336	7.121	6.830
PCB 180 female	6.669	6.767	6.911
PCB 180 male	7.329	7.137	6.823
PCB 183^a^ female	6.375	6.845	7.095
PCB 183^a^ male	7.354	7.087	6.847
PCB 187 female	6.737	6.778	6.841
PCB 187 male	7.287	7.127	6.913
PCB Group 1^b^ female	6.538	6.802	6.975
PCB Group 1^b^ male	7.317	7.112	6.889
PCB Group 2^c^ female	6.531	6.790	7.009
PCB Group 2^c^ male	7.335	7.113	6.845

Robust linear regression models with the FT3 serum concentration in the child at age 6 months as a dependent variable and the respective compound of interest as independent variables reveal lower FT3 concentrations for higher compound concentrations. Analyses were adjusted for maternal serum lipid concentration and an interaction term for the sex of the child was included.

The underlying model reached the level of significance for PCB 99 for females and for PCB 183 for females.

*PCB* polychlorinated biphenyl, *DDE* dichlorodiphenyldichloroethylene, *FT3* free triiodothyronine (pmol/l).

^a^For PCB 183, a polynomial of second order with FT3 serum concentration of the child at age 6 months as dependent variable reveals significant higher FT3 serum concentrations for girls of mothers with higher compound serum concentrations. We checked if a term of higher order was necessary by modelling the associations with polynomial and compare them to the linear model using analysis of variance. If the higher-order term resulted in a significant model improvement, the results were changed accordingly.

^b^Group 1: sum of potentially antiestrogenic and dioxin-like PCB congeners (74 + 118 + 138 + 156 + 170).

^c^Group 2: sum of phenobarbital, CYP1A and CYP2B inducing PCB congeners (99 + 153 + 180 + 183).

**Table 6:** Estimates of maternal FT3 serum concentration for the 10th, 50th and 90th percentile of maternal serum compound concentration.

Independent variable	Maternal FT3 serum concentration (pmol/l) for the		
	10th compound percentile	50th compound percentile	90th compound percentile
DDE^a^	4.361	4.240	4.033
PCB 74	4.283	4.260	4.144
PCB 99	4.286	4.242	4.156
PCB 118	4.328	4.251	4.10
PCB 138	4.353	4.251	4.069
PCB 153	4.349	4.249	4.066
PCB 156	4.307	4.239	4.098
PCB 170	4.311	4.241	4.102
PCB 180^a^	4.374	4.227	4.021
PCB 183	4.370	4.257	4.046
PCB 187	4.353	4.258	4.053
PCB Group 1^b^	4.355	4.251	4.071
PCB Group 2^c^	4.340	4.251	4.075

Robust linear regression models with the maternal FT3 serum concentration as a dependent variable and the respective compound of interest as independent variables reveal lower FT3 concentrations for higher compound concentrations. Analyses were adjusted for maternal age, maternal BMI and maternal serum lipid concentration.

The underlying model reached the level of significance for all compounds and both PCB groups, except PCB 156.

*PCB* polychlorinated biphenyl, *DDE* dichlorodiphenyldichloroethylene, *FT3* free triiodothyronine (pmol/l).

^a^For DDE and PCB 180, a polynomial of second order with FT3 serum concentration as a dependent variable reveals significant higher FT3 serum concentrations for women with higher compound serum concentrations. We checked if a term of higher order was necessary by modelling the associations with polynomial and compare them to the linear model using analysis of variance. If the higher-order term resulted in a significant model improvement, the results were changed accordingly. Otherwise, the previous result was kept.

^b^Group 1: sum of potentially antiestrogenic and dioxin-like PCB congeners (74 + 118 + 138 + 156 + 170).

^c^Group 2: sum of phenobarbital, CYP1A and CYP2B inducing PCB congeners (99 + 153 + 180 + 183).

## Discussion

Although the exact threshold for the endocrine modulatory action of POPs remains unclear, it has been shown that PCBs and *p*,*p*′-DDE are able to cross the placental barrier.^28^ The high percentage of PCB and *p*,*p*′-DDE concentrations above the certified reference concentration in maternal sera from our cohort strongly suggests that both the mothers and their unborn children were exposed to potentially harmful concentrations of POPs, and that our study is, therefore, useful in determining potential adverse effects of POPs.

Regarding the demographic characteristics that could potentially influence the maternal serum concentration of POPs, we found age and maternal BMI to be the most influential. Here, we observed significant associations between age and all tested compounds, except PCB 74, and between BMI and PCB 153, 156, 170, 180 and 187. This finding is likely attributable to the rise in lipophilicity-induced accumulation in human adipose tissue as the degree of chlorination increases. Both findings are in line with a previous study.^29^

Another major finding of our study was significant positive associations between maternal POP levels and postnatal weight development, with a significant association between specific PCBs (99, 118, 138, 153, 156, 170, 180, Group 1, Group 2) and *p*,*p*′-DDE and child weight gain. PCB effects on weight have been reported in multiple prior studies as ranging from no effect to positive or even negative correlations.^30, 31^ While the reasons for these varying results remain elusive, the mechanisms through which PCBs might affect weight gain are presumably a combination of effects on the action of thyroid hormones,^32^ oestrogen-related receptor γ^33^ and glucose homeostasis,^34^ all factors that critically influence postnatal development. As weight gain within the first 2 years of life is known to be linked to metabolic alterations in adulthood, these findings suggest a potential influence of prenatal POP exposure on the development of metabolic diseases such as type 2 diabetes later in life.

Other anthropometric parameters for the children in our cohort, including weight and head circumference at birth, were not associated with maternal POP levels with the exception of PCB 183, which was negatively associated with the child’s length at birth. These findings only partially support the results of the Spanish INMA (INfancio y Medio Ambiente) cohort, which showed a significant reduction of foetal femur length from week 20 onward in individuals exposed to higher PCB levels.^35^ Other studies did not confirm a significant association between PCBs and length at birth.^36^

Our study additionally revealed a potential impact of POPs on the thyroid hormone status in mothers and their children. We found a negative correlation between *p*,*p*′-DDE and all PCB levels, except PCB 156, and FT3 levels in mothers, and a negative correlation between *p*,*p*′-DDE, PCB 74 and 118 exposure and maternal TSH levels. Moreover, we detected a positive association between maternal PCB 99 and 183 and FT3 in the respective female children at an age of 6 months, whereas we found a negative association between *p*,*p*′-DDE and FT3 serum levels in boys at an age of 1 year. The inverse association between PCBs and FT3 serum levels in maternal blood has been reported on more than one occasion in prior research,^37, 38^ although other studies report different results,^39^ with no association or even positive correlations between POP levels and thyroid hormone parameters.^25^ These discrepancies might be due to differences in the size or sex distribution of the study cohorts, concentration ranges of the POPs investigated or differences in the covariables used. Takser et al.,^38^ who also observed a negative association between maternal serum levels for PCB 138, 153 and 180 and maternal T3 serum levels, found no significant association between maternal PCB exposure and the child’s thyroid hormone levels. The difference in PCB serum concentration, which was twice as high in our cohort, might be responsible for the discrepancy in these findings. Wilhelm et al.^39^ similarly found no impairment of the thyroid function of infants, although the PCB concentrations they measured were three times higher, which leads to the assumption that the discrepancy in the findings cannot solely be explained by differences in the compound concentrations. Although this variance in findings has not yet been fully explained, and may be due to as yet unidentified factors, the influence of certain PCBs and *p*,*p*′-DDE on the thyroid hormone system seems likely. The underlying mechanisms of the potential influence of POPs on FT3 and TSH levels might include an increase in the hepatic metabolism of thyroid hormones resulting from exposure of the liver to POP compounds.^40^ However, this would not explain the observed decrease in maternal TSH. Another possible explanation is therefore a potential direct effect on the action of thyroid hormones via interaction with the thyroid hormone receptor, which might trigger a negative feedback in the pituitary gland, resulting in a decrease in TSH and a consequent decrease in FT3.^25^

One strength of our study is the simultaneous availability of blood samples from mothers and their children, as well as the wide range of measured child characteristics. These could potentially be used for a follow-up study looking at the impact of PCBs and *p*,*p*′-DDE on development later in life. The limitations include the relatively small sample size and the isolated analysis of individual compounds, compared with other studies, which focused on compound mixtures. Other limitations include the relatively small effect sizes, which, in combination with multiple comparisons, increases the risk of type one error. In concert with related studies, however, the observed effects contribute to a better understanding of the potential mode of action of POPs and their harmful effects in humans. The significant associations between the analysed compounds and certain parameters of child development and thyroid hormone status support the assertion that further research is needed on the potentially harmful action of PCBs and *p*,*p*′-DDT/*p*,*p*′-DDE.

## Supplementary information

Supplementary Tables
