# Supplementary material for: Persistent organic pollutants in pregnant women potentially affect child development and thyroid hormone status
Source: Pediatr Res. 2021 Apr 6;91(3):690–8. doi: 10.1038/s41390-021-01488-5 (PMC8904258; doi:10.1038/s41390-021-01488-5)
Supplement: Supplementary file 1 — Supplementary Tables [file 41390_2021_1488_MOESM1_ESM.docx]

| Model 2a (n=218) dependent variable= duration of pregnancy (days) | | | | | | | |
| --- | --- | --- | --- | --- | --- | --- | --- |
| independent variable | | Beta | | Std.Error | | Pr(>\|t\|) | |
| DDE | | -0.0001 | | 0.0002 | | 0.553 | |
| PCB 74 | | 0.006 | | 0.023 | | 0.793 | |
| PCB 99 | | -0.428 | | 2.704 | | 0.874 | |
| PCB 99 poly 2^a^ | | -0.714 | | 3.244 | | 0.826 | |
| PCB 118 | | 0.003 | | 0.011 | | 0.741 | |
| PCB 138 | | -0.0008 | | 0.003 | | 0.782 | |
| PCB 153 | | -0.0002 | | 0.002 | | 0.933 | |
| PCB 156 | | 0.004 | | 0.015 | | 0.775 | |
| PCB 170 | | 0.001 | | 0.005 | | 0.828 | |
| PCB 180 | | 0.0003 | | 0.003 | | 0.895 | |
| PCB 183 | | -0.013 | | 0.017 | | 0.417 | |
| PCB 187 | | -0.004 | | 0.010 | | 0.668 | |
| PCB group 1^b^ | | 2.51x 10^-05^ | | 0.001 | | 0.986 | |
| PCB group 2^c^ | | -4.7x 10^-05^ | | 0.0010 | | 0.962 | |
| Model 2b(n=194) dependent variable= head circumference at birth (cm) | | | | | | | |
| independent variable | Beta | | | Std.Error | | | Pr(>\|t\|) |
| DDE | -0.0004 | | | 0.0003 | 0.117 | | |
| PCB 74 | -0.005 | | | 0.027 | 0.863 | | |
| PCB 99 | -0.009 | | | 0.026 | 0.737 | | |
| PCB 118 | 0.004 | | | 0.011 | 0.752 | | |
| PCB 138 | -0.0008 | | | 0.003 | 0.783 | | |
| PCB 153 | 0.0001 | | | 0.002 | 0.957 | | |
| PCB 156 | 0.014 | | | 0.015 | 0.346 | | |
| PCB 170 | 0.004 | | | 0.005 | 0.463 | | |
| PCB 180 | 0.002 | | | 0.003 | 0.461 | | |
| PCB 183 | -0.027 | | | 0.021 | 0.194 | | |
| PCB 187 | -0.003 | | | 0.010 | 0.790 | | |
| PCB group 1^b^ | 0.0003 | | | 0.001 | 0.829 | | |
| PCB group 2^c^ | 0.0002 | | | 0.001 | 0.812 | | |
| Model 2c (n=217) dependent variable= body length at birth (cm) | | | | | | | |
| independent variable | | Beta | | Std.Error | Pr(>\|t\|) | | |
| DDE | | -0.0002 | | 0.0004 | 0.701 | | |
| PCB 74 | | -0.019 | | 0,039 | 0.630 | | |
| PCB 99 | | -0.050 | | 0.049 | 0.314 | | |
| PCB 118 | | -0.011 | | 0.017 | 0.526 | | |
| PCB 138 | | -0.007 | | 0.005 | 0.173 | | |
| PCB 153 | | -0.004 | | 0.003 | 0.213 | | |
| PCB 156 | | -0.029 | | 0.026 | 0.275 | | |
| PCB 170 | | -0.009 | | 0.008 | 0.295 | | |
| PCB 180 | | -0.005 | | 0.005 | 0.240 | | |
| PCB 183 | | -0.060 | | 0.028 | 0.031 | | |
| PCB 187 | | -0.029 | | 0.018 | 0.110 | | |
| PCB group 1^b^ | | -0.003 | | 0.003 | 0.225 | | |
| PCB group 2^c^ | | -0.002 | | 0.002 | 0.201 | | |
|  | | | | | | | |
| Model 2d (n=217) dependent variable= bodyweight at birth (g) | | | | | | | |
| Independent variable | | | Beta | Std.Error | Pr(>\|t\|) | | |
| DDE | | | -0.123 | 0.083 | 0.140 | | |
| PCB 74 | | | -1.310 | 8.282 | 0.874 | | |
| PCB 99 | | | -9.783 | 8.722 | 0.263 | | |
| PCB 118 | | | -1.105 | 3.811 | 0.772 | | |
| PCB 138 | | | -1.509 | 0.993 | 0.130 | | |
| PCB 153 | | | -0.798 | 0.615 | 0.196 | | |
| PCB 156 | | | -4.865 | 5.129 | 0.344 | | |
| PCB 170 | | | -1.586 | 1.694 | 0.350 | | |
| PCB 180 | | | -0.903 | 0.913 | 0.324 | | |
| PCB 183 | | | -9.883 | 6.166 | 0.110 | | |
| PCB 187 | | | -5.054 | 3.465 | 0.146 | | |
| PCB group 1^b^ | | | -0.595 | 0.495 | 0.230 | | |
| PCB group 2^c^ | | | -0.430 | 0.346 | 0.216 | | |

|  | | | | |
| --- | --- | --- | --- | --- |
| Model 2e (n=179) dependent variable= child’s weight gain in the first year of life (%) | | | | |
| Independent variable | Beta | Std.Error | Pr(>\|t\|) |  |
| DDE | 0.006 | 0.007 | 0.408 |  |
| PCB 74 | 0.841 | 0.671 | 0.212 |  |
| PCB 99 | 1.166 | 0.916 | 0.205 |  |
| PCB 118 | 0.579 | 0.308 | 0.062 |  |
| PCB 138 | 0.153 | 0.083 | 0.066 |  |
| PCB 153 | 0.095 | 0.051 | 0.066 |  |
| PCB 156 | 0.797 | 0.440 | 0..072 |  |
| PCB 170 | 0.255 | 0.139 | 0.067 |  |
| PCB 180 | 0.132 | 0.077 | 0.087 |  |
| PCB 183 | 0.245 | 0.585 | 0.676 |  |
| PCB 187 | 0.240 | 0.336 | 0.476 |  |
| PCB group 1^b^ | 0.081 | 0.040 | 0.045 |  |
| PCB group 2^c^ | 0.049 | 0.038 | 0.199 |  |

| Model 2f (n=153) dependent variable= child’s weight gain in the first two years of life (%) | | | | |
| --- | --- | --- | --- | --- |
| Independent variable | Beta | Std.Error | Pr(>\|t\|) |  |
| DDE | 0.017 | 0.008 | 0.029 |  |
| PCB 74 | 0.733 | 0.823 | 0.375 |  |
| PCB 99 | 2.976 | 0.999 | 0.003 |  |
| PCB 118 | 0.873 | 0.418 | 0.038 |  |
| PCB 138 | 0.301 | 0.111 | 0.008 |  |
| PCB 153 | 0.172 | 0.069 | 0.014 |  |
| PCB 156 | 1.323 | 0.545 | 0.016 |  |
| PCB 170 | 0.424 | 0.189 | 0.026 |  |
| PCB 180 | 0.207 | 0.10 | 0.040 |  |
| PCB 183 | 1.158 | 0.690 | 0.095 |  |
| PCB 187 | 0.522 | 0.386 | 0.179 |  |
| PCB group 1^b^ | 0.143 | 0.057 | 0.013 |  |
| PCB group 2^c^ | 0.093 | 0.039 | 0.019 |  |

**Table S1 PCB and p,p’-DDE maternal serum concentrations and child characteristics. Robust linear regression model**

Table S1 shows the results of a robust linear regression model with a child characteristic as dependent variable and the compounds of interest, and two groups of compounds, as independent variables. We included maternal age, maternal BMI and maternal serum lipids as independent covariates for all models. For model 2d-f we additionally included maternal nicotine consumption as independent covariate.

-The beta coefficient of the Model 2a shows the change of the duration of pregnancy in days per unit change of maternal serum concentration of the compound in (pg/ml)

-The beta coefficient of the Models 2b and c show the change of head circumference at birth and length at birth in cm per unit change of maternal serum concentration of the compound in (pg/ml)

-The beta coefficient of the Model 2d show the change of weight at birth in g per unit change of maternal serum concentration of the compound in (pg/ml)

-The beta coefficient of the Model 2e and f show the weight gain in the first 2 years of life in % per unit change of maternal serum concentration of the compound in (pg/ml)

Pr(>|t|) = p-value for the t-test; Std. Error= Standard Error

PCB: Polychlorinated biphenyl

DDE: Dichlorodiphenyldichloroethylene

a: if the model showed significant improvement using a terms of higher order, the second order term was included

We checked if a terms of higher order were necessary by modeling the associations with polynomial and compare them to the linear model using analysis of variance. If the higher order term resulted in a significant model improvement the results were changed accordingly. Otherwise, the previous result was kept.

b: Group 1: sum of potentially antiestrogenic and dioxin-like PCB congeners (74+118+138+156+170)

c: Group 2: sum of Phenobarbital, CYP1A and CYP2B inducing PCB congeners (99+ 153+180+183)

| \|  \| \| \| \| \| \| \| --- \| --- \| --- \| --- \| --- \| --- \| \| Model 3a (n=93) Dependent variable= child’s TSH serum concentration at age 6 months (mU/l) \| \| \| \| \| \| \| \| independent variable \| Beta \| Std.Error \| Pr(>\|t\|) \| \| DDE \| -0.0003 \| 0.0003 \| 0.351 \| \| PCB 74 \| -0.14 \| 0.094 \| 0.139 \| \| PCB 74 poly 2^a^ \| 0.005 \| 0.003 \| 0.102 \| \| PCB 99 \| -0.196 \| 0.091 \| 0.033 \| \| PCB 99 poly 2^a^ \| 0.007 \| 0.003 \| 0.018 \| \| PCB 118 \| -0.114 \| 0.046 \| 0.015 \| \| PCB 118 poly2^a^ \| 0.002 \| 0.0008 \| 0.015 \| \| PCB 138 \| 0.001 \| 0.003 \| 0.765 \| \| PCB 153 \| 0.001 \| 0.002 \| 0.724 \| \| PCB 156 \| 0.005 \| 0.015 \| 0.738 \| \| PCB 170 \| 0.002 \| 0.005 \| 0.733 \| \| PCB 180 \| 0.0007 \| 0.002 \| 0.781 \| \| PCB 183 \| 0.017 \| 0.018 \| 0.351 \| \| PCB 187 \| 0.004 \| 0.010 \| 0.679 \| \| PCB group 1^b^ \| 0.0004 \| 0.001 \| 0.80 \| \| PCB group 2^c^ \| 0.0004 \| 0.001 \| 0.712 \| \| Model 3b(N=92) Dependent variable= child’s FT3 serum concentration at age 6 months (pmol/l) \| \| \| \| \| \| independent variable \| Beta \| Std.Error \| Pr(>\|t\|) \| \| DDE \| 0.0002 \| 0.0003 \| 0.445 \| \| PCB 74 \| -0.035 \| 0.039 \| 0.377 \| \| PCB 99 female \| 0.061 \| 0.025 \| 0.016 \| \| PCB 99 male \| -0.004 \| 0.019 \| 0.847 \| \| PCB 118 \| -0.006 \| 0.013 \| 0.618 \| \| PCB 138 \| 0.004 \| 0.003 \| 0.201 \| \| PCB 153 \| 0.002 \| 0.003 \| 0.490 \| \| PCB 156 \| 0.011 \| 0.017 \| 0.501 \| \| PCB 170 \| 0.006 \| 0.005 \| 0.229 \| \| PCB 180 \| 0.002 \| 0.003 \| 0.491 \| \| PCB 183 \| 0.042 \| 0.026 \| 0.111 \| \| PCB 183 female \| 0.156 \| 0.058 \| 0.009 \| \| PCB 183 female poly 2 \| -0.004 \| 0.002 \| 0.007 \| \| PCB 183 male \| -0.08 \| 0.045 \| 0.078 \| \| PCB 183 male poly 2 \| 0.002 \| 0.001 \| 0.135 \| \| PCB 187 \| 0.005 \| 0.010 \| 0.637 \| \| PCB group 1^b^ \| 0.0007 \| 0.001 \| 0.604 \| \| PCB group 2^c^ \| 0.0005 \| 0.0009 \| 0.595 \|  \|  \| \| \| \| \| --- \| --- \| --- \| --- \| \| independent variable \| Beta \| Std.Error \| Pr(>\|t\|) \| \| DDE \| -0.0003 \| 0.0005 \| 0.508 \| \| PCB 74 poly 1 \| -0.251 \| 0.175 \| 0.155 \| \| PCB 74 poly 2^a^ \| 0.01 \| 0.006 \| 0.093 \| \| PCB 99 \| -0.016 \| 0.052 \| 0.753 \| \| PCB 118 \| -0.007 \| 0.024 \| 0.758 \| \| PCB 138 \| -0.006 \| 0.006 \| 0.308 \| \| PCB 153 \| -0.002 \| 0.003 \| 0.502 \| \| PCB 156 \| -0.010 \| 0.027 \| 0.699 \| \| PCB 170 \| -0.004 \| 0.009 \| 0.610 \| \| PCB 180 \| -0.0008 \| 0.005 \| 0.863 \| \| PCB 183 \| -0.035 \| 0.032 \| 0.271 \| \| PCB 187 \| -0.020 \| 0.017 \| 0239 \| \| PCB group 1^b^ \| -0.002 \| 0.003 \| 0.431 \| \| PCB group 2^c^ \| -0.0009 \| 0.002 \| 0.611 \| \| Model 3d(n=81) Dependent variable= child’s TSH serum concentration at age 1 year (mU/l) \| \| \| \| \| independent variable \| Beta \| Std.Error \| Pr(>\|t\|) \| \| DDE \| -0.0003 \| 0.0003 \| 0.312 \| \| PCB 74 \| -0.070 \| 0.038 \| 0.072 \| \| PCB 99 \| -0.049 \| 0.029 \| 0.097 \| \| PCB 118 \| -0.028 \| 0.014 \| 0.050 \| \| PCB 138 \| -0.005 \| 0.003 \| 0.136 \| \| PCB 153 \| -0.003 \| 0.002 \| 0.096 \| \| PCB 156 \| -0.028 \| 0.017 \| 0.111 \| \| PCB 170 \| -0.009 \| 0.005 \| 0.086 \| \| PCB 180 \| -0.005 \| 0.003 \| 0.085 \| \| PCB 183 \| -0.033 \| 0.022 \| 0.143 \| \| PCB 187 \| -0.018 \| 0.011 \| 0.114 \| \| PCB group 1^b^ \| -0.003 \| 0.002 \| 0.101 \| \| PCB group 2^c^ \| -0.002 \| 0.001 \| 0.101 \|  \| Model 3e(n=86) Dependent variable =child’s FT3 serum concentration at age 1year (pmol/l) \| \| \| \| \| \| --- \| --- \| --- \| --- \| --- \| \| independent variable \| Beta \| Estimate \| Pr(>\|t\|) \| \| DDE male \| -0.0006 \| 0.0002 \| 0.018 \| \| DDE female \| 6.9 x 10^-5^ \| 0.0003 \| 0.809 \| \| PCB 74 \| 0.023 \| 0.035 \| 0.507 \| \| PCB 99 \| -0.010 \| 0.025 \| 0.706 \| \| PCB 118 \| 0.006 \| 0.012 \| 0.602 \| \| PCB 138 \| -0.006 \| 0.004 \| 0.075 \| \| PCB 153 \| -0.004 \| 0.002 \| 0.059 \| \| PCB 156 \| -0.036 \| 0.018 \| 0.054 \| \| PCB 170 \| -0.012 \| 0.006 \| 0.057 \| \| PCB 180 \| -0.006 \| 0.003 \| 0.065 \| \| PCB 183 \| -0.025 \| 0.023 \| 0.281 \| \| PCB 187 \| -0.009 \| 0.012 \| 0.460 \| \| PCB group 1^b^ \| -0.003 \| 0.002 \| 0.115 \| \| PCB group 2^c^ \| -0.002 \| 0.001 \| 0.065 \|  \| Model 3f(n=85) Dependent variable=child’s FT4 serum concentration at age 1 year (pmol/l) \| \| \| \| \| \| --- \| --- \| --- \| --- \| --- \| \| independent variable \| Beta \| Std.Error \| Pr(>\|t\|) \| \| DDE \| -0.0005 \| 0.0006 \| 0.429 \| \| PCB 74 \| -0.120 \| 0.086 \| 0.165 \| \| PCB 74 poly 1 \| -0.682 \| 0.349 \| 0.054 \| \| PCB 74 poly 2^a^ \| 0.031 \| 0.015 \| 0.044 \| \| PCB 99 \| -0.035 \| 0.066 \| 0.594 \| \| PCB 118 \| -0.005 \| 0.037 \| 0.892 \| \| PCB 138 \| -0.002 \| 0.008 \| 0.802 \| \| PCB 153 \| -0.0008 \| 0.004 \| 0.860 \| \| PCB 156 \| -0.012 \| 0.040 \| 0.763 \| \| PCB 170 \| 0.003 \| 0.0116 \| 0.827 \| \| PCB 180 \| 0.002 \| 0.006 \| 0.801 \| \| PCB 183 \| 0.027 \| 0.037 \| 0.465 \| \| PCB 187 \| 0.016 \| 0.025 \| 0.539 \| \| PCB group 1^b^ \| -0.0003 \| 0.004 \| 0.931 \| \| PCB group 2^c^ \| 0.0002 \| 0.002 \| 0.945 \|   **Table S2 PCB and p,p’-DDE maternal serum concentrations and child’s thyroid hormone concentrations at 6 months and 1 year. Robust linear regression model**  Table S2 shows the results of a robust linear regression model with a thyroid hormone parameter in the child as dependent variable and the compounds of interest, and two groups of compounds, as independent variables. We included maternal serum lipids as independent covariate. Furthermore, we included an interaction term to analyse interaction between the compounds and the sex of the child. If the interaction was significant, the effect was reported for male and female separately.  The beta coefficient of the Models 3a and d show the change of the TSH serum concentration of the child in (mU/l) per change of unit maternal serum compound concentration in (pg/ml)  The beta coefficient of the Models 3b-c and 3e-f show the change of the FT3 and FT4 serum concentration of the child in pmol/l per change of unit maternal serum compound concentration in (pg/ml)  Model 3b: if the interaction term was significant, the effect was reported for male and female separately  Pr(>\|t\|) = p-value for the t-test; Std. Error= Standard Error  PCB: Polychlorinated biphenyl  DDE: Dichlorodiphenyldichloroethylene  a: if the model showed significant improvement using a terms of higher order, the second order term was included  We checked if a terms of higher order were necessary by modeling the associations with polynomial and compare them to the linear model using analysis of variance. If the higher order term resulted in a significant model improvement the results were changed accordingly. Otherwise, the previous result was kept.  b: Group 1: sum of potentially antiestrogenic and dioxin-like PCB congeners (74+118+138+156+170)  c: Group 2: sum of Phenobarbital, CYP1A and CYP2B inducing PCB congeners (99+ 153+180+183) |
| --- | --- | --- | --- | --- | --- | --- | --- | --- | --- | --- | --- | --- | --- | --- | --- | --- | --- | --- | --- | --- | --- | --- | --- | --- | --- | --- | --- | --- | --- | --- | --- | --- | --- | --- | --- | --- | --- | --- | --- | --- | --- | --- | --- | --- | --- | --- | --- | --- | --- | --- | --- | --- | --- | --- | --- | --- | --- | --- | --- | --- | --- | --- | --- | --- | --- | --- | --- | --- | --- | --- | --- | --- | --- | --- | --- | --- | --- | --- | --- | --- | --- | --- | --- | --- | --- | --- | --- | --- | --- | --- | --- | --- | --- | --- | --- | --- | --- | --- | --- | --- | --- | --- | --- | --- | --- | --- | --- | --- | --- | --- | --- | --- | --- | --- | --- | --- | --- | --- | --- | --- | --- | --- | --- | --- | --- | --- | --- | --- | --- | --- | --- | --- | --- | --- | --- | --- | --- | --- | --- | --- | --- | --- | --- | --- | --- | --- | --- | --- | --- | --- | --- | --- | --- | --- | --- | --- | --- | --- | --- | --- | --- | --- | --- | --- | --- | --- | --- | --- | --- | --- | --- | --- | --- | --- | --- | --- | --- | --- | --- | --- | --- | --- | --- | --- | --- | --- | --- | --- | --- | --- | --- | --- | --- | --- | --- | --- | --- | --- | --- | --- | --- | --- | --- | --- | --- | --- | --- | --- | --- | --- | --- | --- | --- | --- | --- | --- | --- | --- | --- | --- | --- | --- | --- | --- | --- | --- | --- | --- | --- | --- | --- | --- | --- | --- | --- | --- | --- | --- | --- | --- | --- | --- | --- | --- | --- | --- | --- | --- | --- | --- | --- | --- | --- | --- | --- | --- | --- | --- | --- | --- | --- | --- | --- | --- | --- | --- | --- | --- | --- | --- | --- | --- | --- | --- | --- | --- | --- | --- | --- | --- | --- | --- | --- | --- | --- | --- | --- | --- | --- | --- | --- | --- | --- | --- | --- | --- | --- | --- | --- | --- | --- | --- | --- | --- | --- | --- | --- | --- | --- | --- | --- | --- | --- | --- | --- | --- | --- | --- | --- | --- | --- | --- | --- | --- | --- | --- | --- | --- | --- | --- | --- | --- | --- | --- | --- | --- | --- | --- | --- | --- | --- | --- | --- | --- | --- | --- | --- | --- | --- | --- | --- | --- | --- | --- | --- | --- | --- | --- | --- | --- | --- | --- | --- | --- | --- | --- | --- | --- | --- | --- | --- | --- | --- | --- | --- | --- | --- | --- | --- | --- | --- | --- | --- | --- | --- | --- | --- | --- | --- | --- | --- | --- | --- | --- | --- | --- | --- | --- | --- | --- | --- | --- | --- | --- | --- | --- | --- | --- | --- | --- | --- | --- | --- | --- | --- | --- | --- | --- | --- | --- |

|  | | | | | | | | | |
| --- | --- | --- | --- | --- | --- | --- | --- | --- | --- |
| \| \| \| Model 4a (n=231) Dependent variable=maternal TSH serum concentration (mU/l) \| \| --- \| \| \| --- \| --- \| \| \| --- \| --- \| --- \| | | | | | | | | |  |
| Independent variable | | Beta | | Std.Error | | Pr(>\|t\|) | | |  |
| DDE poly 1 | | -1.6x 10x^-03^ | | 7.1 x 10^-04^ | | 0.020 | | |  |
| DDE poly 2^a^ | | 1.1x10^-06^ | | 3.4 x 10^-07^ | | 0.002 | | |  |
| PCB 74 poly 1 | | -0.072 | | 0.067 | | 0.280 | | |  |
| PCB 74 poly 2^a^ | | 0.005 | | 0.002 | | 0.007 | | |  |
| PCB 99 | | -0.015 | | 0.013 | | 0.248 | | |  |
| PCB 118 | | -0.012 | | 0.006 | | 0.043 | | |  |
| PCB 138 | | -0.002 | | 0.002 | | 0.211 | | |  |
| PCB 153 | | -0.001 | | 0.001 | | 0.247 | | |  |
| PCB 156 | | -0.007 | | 0.009 | | 0.431 | | |  |
| PCB 170 | | -0.001 | | 0.003 | | 0.608 | | |  |
| PCB 180 | | -0.0005 | | 0.001 | | 0.709 | | |  |
| PCB 183 | | -0.005 | | 0.009 | | 0.626 | | |  |
| PCB 187 | | -0.004 | | 0.005 | | 0.470 | | |  |
| PCB group 1^a^ | | -0.0009 | | 0.0008 | | 0.245 | | |  |
| PCB group 2^b^ | | -0.0005 | | 0.0006 | | 0.380 | | |  |
| \| \| Model 4b (n=224) Dependent variable=maternal FT3 serum concentration (pmol/l) \| \| --- \| \| \| --- \| --- \| | | | | | | | |  |  |
| independent variable | Beta | | Std.Error | | Pr(>\|t\|) | |  |  |  |
| DDE | -0.0007 | | 0.0003 | | 0.002 | |  |  |  |
| DDE poly 2^a^ | 3.2 x 10^-07^ | | 1.2 x 10^-07^ | | 0.011 | |  |  |  |
| PCB 74 | -0.024 | | 0.008 | | 0.005 | |  |  |  |
| PCB 99 | -0.018 | | 0.008 | | 0.016 | |  |  |  |
| PCB 118 | -0.012 | | 0.003 | | 0.0007 | |  |  |  |
| PCB 138 | -0.003 | | 0.001 | | 0.006 | |  |  |  |
| PCB 153 | -0.002 | | 0.0007 | | 0.006 | |  |  |  |
| PCB 156 | -0.011 | | 0.006 | | 0.057 | |  |  |  |
| PCB 170 | -0.004 | | 0.002 | | 0.042 | |  |  |  |
| PCB 180 | -0.008 | | 0.003 | | 0.003 | |  |  |  |
| PCB 180 poly 2^a^ | 2.3x 10^-05^ | | 9.6x 10^-06^ | | 0.018 | |  |  |  |
| PCB 183 | -0.021 | | 0.006 | | 0.0003 | |  |  |  |
| PCB 187 | -0.011 | | 0.003 | | 0.003 | |  |  |  |
| PCB group 1^b^ | -0.002 | | 0.0005 | | 0.005 | |  |  |  |
| PCB group 2^c^ | -0.001 | | 0.0004 | | 0.010 | |  |  |  |

| \| Model 4c (n=227) Dependent variable=maternal FT4 serum concentration (pmol/l) \| \| --- \| | | | | |
| --- | --- | --- | --- | --- | --- |
| independent variable | Beta | Std.Error | Pr(>\|t\|) |  |
| DDE | 4.27x 10^-05^ | 0.0002 | 0.863 |  |
| PCB 74 | 0.028 | 0.026 | 0.283 |  |
| PCB 99 | 0.012 | 0.026 | 0.628 |  |
| PCB 118 | 0.003 | 0.011 | 0.784 |  |
| PCB 138 | -0.0003 | 0.003 | 0.929 |  |
| PCB 153 | -0.0008 | 0.002 | 0.708 |  |
| PCB 156 | -0.016 | 0.018 | 0.385 |  |
| PCB 170 | -0.007 | 0.006 | 0.194 |  |
| PCB 180 | -0.003 | 0.003 | 0.258 |  |
| PCB 183 | -0.011 | 0.018 | 0.553 |  |
| PCB 187 | -0.011 | 0.011 | 0.316 |  |
| PCB group 1^b^ | -0.0007 | 0.002 | 0.669 |  |
| PCB group 2^c^ | -0.0008 | 0.001 | 0.505 |  |

**Table S3 PCB and p,p’-DDE maternal serum concentrations and thyroid hormone concentrations of the mother at gestational age 24 weeks. Robust linear regression model**

Table S3 shows the results of a robust linear regression model with a thyroid hormone parameter in the mother as dependent variable and the compounds of interest, and two groups of compounds, as independent variables. We included maternal age, maternal BMI and maternal serum lipids as independent covariates.

The beta coefficient of the Model 4a show the change of the TSH serum concentration in (mU/l) per change of unit maternal serum compound concentration in (pg/ml)

The beta coefficient of the Models 4b-c show the change of the FT3 and FT4 serum concentration in pmol/l per change of unit maternal serum compound concentration in (pg/ml)

Pr(>|t|) = p-value for the t-test; Std. Error= Standard Error

PCB: Polychlorinated biphenyl

DDE: Dichlorodiphenyldichloroethylene

a: if the model showed significant improvement using a terms of higher order, the second order term was included

We checked if a terms of higher order were necessary by modeling the associations with polynomial and compare them to the linear model using analysis of variance. If the higher order term resulted in a significant model improvement the results were changed accordingly. Otherwise, the previous result was kept.

b: Group 1: sum of potentially antiestrogenic and dioxin-like PCB congeners (74+118+138+156+170)

c: Group 2: sum of Phenobarbital, CYP1A and CYP2B inducing PCB congeners (99+ 153+180+183)
